# Elevated levels of perfluoroalkyl acids in family members of occupationally exposed workers: the importance of dust transfer

**DOI:** 10.1038/srep09313

**Published:** 2015-03-20

**Authors:** Jianjie Fu, Yan Gao, Thanh Wang, Yong Liang, Aiqian Zhang, Yawei Wang, Guibin Jiang

**Affiliations:** 1 State Key Laboratory of Environmental Chemistry and Ecotoxicology, Research Center for Eco-Environmental Sciences, Chinese Academy of Sciences, Beijing 100085, China; 2 Jianghan University, Wuhan, Hubei, China

## Abstract

The exposure pathways of perfluoroalkyl acids (PFAAs) to humans are still not clear because of the complex living environment, and few studies have simultaneously investigated the bioaccumulative behaviour of different PFAAs in humans. In this study, serum, dust, duplicate diet, and other matrices were collected around a manufacturing plant in China, and homologous series of PFAAs were analysed. PFAA levels in dust and serum of local residents in this area were considerably higher than those in non-polluted area. Although dietary intake was the major exposure pathway in the present study, dust ingestion played an important role in this case. Serum PFAAs in local residents was significantly correlated with dust PFAAs levels in their living or working microenvironment. Serum PFAAs and dust PFAAs were significantly higher in family members of occupational workers (FM) than in ordinary residents (OR) (p < 0.01). After a careful analysis of the PFAAs exposure pathway, a potential pathway in addition to direct dust ingestion was suggested: PFAAs might transferred from occupational worker's clothes to dinners via cooking processes. The bioaccumulative potential of PFHxS and PFOS were higher than other PFAAs, which suggested a substantial difference between the bioaccumulative ability of perfluorinated sulfonic acids and perfluorinated carboxylic acids.

Perfluoroalkyl acids (PFAAs) have been extensively used as surfactants, lubricants, stain- and water-resistant coatings, and fire-resistant foams, due to their unique properties^1^. Perfluorooctane sulfonic acid (PFOS) and perfluorooctanoic acid (PFOA) are the most widely distributed PFAAs in the world, and they have attracted increasing concern in the past decade^2^,^3^,^4^. The total historical global production of perfluorooctane sulfonyl fluoride (POSF) reached approximately 96,000 tonnes (t) and approximately 45250 t of POSF was emitted into the environment from 1970 to 2012^5^. The primary PFOS producer, 3 M, ceased the production of POSF in 2002. In addition, the production and usage of PFOS, its salts and POSF were restricted by the Stockholm Convention in 2009^6^. However, the production increased rapidly in China in recent years^7^. PFAAs with a fluorinated chain longer than seven are generally considered bioaccumulative, extremely persistent, and have been found to be ubiquitous in the environment^3^,^8^.

PFAAs have been detected in human blood worldwide, and in certain cases, their levels were even higher than those of other persistent organic pollutants^9^,^10^. Recently, the adverse effects to humans associated with PFAAs body burden have been reported, such as osteoarthritis and a later age of puberty^11^,^12^. Humans can be exposed to PFAAs through various pathways^13^,^14^,^15^, but the exposure pathways and bioaccumulative behaviour in humans are still not clear^16^,^17^. Previous studies considered that diet was the primary source^15^,^18^,^19^; however, there might be other significant exposure pathways for PFAAs beside diet^20^. Vestergren et al. reported that the ingestion of house dust was the second most important pathway in the high-exposure scenario^21^, and Beeson et al. ascribed the exceptionally high serum concentrations of some PFAAs in a Canadian family to dust ingestion and/or inhalation^17^.

Our previous study investigated the transportation of PFAAs from the manufacturing facility to the surrounding environment and elevated perfluorohexanesulfonate (PFHxS), PFOS, and PFOA levels in soil and biota samples were found within three kilometres of the plant^22^. Although PFOS production has been restricted by the Stockholm Convention^6^, there is still a usage exemption of PFOS for specific purposes. Thus, the production of PFOS and related products in this particular facility did not immediately halt. The purpose of the present study was to (I) evaluate PFAAs body burden of local residents around this fluorochemical manufacturing facility and to identify the exposure pathways of PFAAs in this area further and (II) to investigate bioaccumulation behaviours of PFAAs with different carbon chain lengths and functional groups in humans. Currently, this paper is the first that intensively studies the exposure pathway of PFAAs to residents near a point source.

## Methods

### Experimental Design and Sampling

A fluorochemical manufacturing plant located in the Hubei province of China was selected as the sampling area. The main synthesis method for PFAAs in this plant is by the electrochemical fluorination process. The sampling procedure was performed in December 2011 and 2012. Fifty-eight local residents who lived around this plant within a distance of 3 km were recruited in the present study. The gender, age, weight, occupation, dietary habits, and other information were collected by questionnaires; the demographic information of studied population is listed in Table S1. In this study, we collected blood samples of each participant to evaluate the PFAAs body burden in humans. According to whether they had family members working in the fluorochemical plant, our studied population, the local residents, were divided into two groups: family members of occupational workers (FM) and ordinary residents (OR). All the experiments (including design and execution) and sampling procedures were carried out in accordance with the guidelines approved by the Medical Research Ethics Committee, School of Medicine, Jianghan University, and all volunteers gave their written informed consent to participate in this study.

Furthermore, to investigate the exposure pathways of PFAAs to residents around the manufacturing facility, relevant exposure matrices (such as dust, Total Suspended Particle (TSP), and duplicate diet) from their living environment were also collected. Dust and TSP samples were collected from the plant and the corresponding participants' residential environment. Duplicate diet and drinking water samples were directly collected from the table and tap from the home of participants. In addition, the work clothes of workers and local residents were also collected. A total of 141 samples, including serum (n = 58), dust (n = 38), TSP (n = 15), drinking water (n = 6), duplicate diet (n = 9) and clothes (n = 15), were collected in this study. The detailed information of these samples (such as sampling sites, number of samples, and grouping of samples) is listed in Table 1, and the spatial distribution of the sampling sites and detailed sampling procedures can be found in the Supplementary Information (Figure S1).

### Chemical Analysis, Quality Assurance, and Quality Control

Eleven PFAAs (perfluoro-n-pentanoic acid (PFPeA), perfluorohexanoic acid (PFHxA), perfluoroheptanoic acid (PFHpA), PFOA, perfluorononanoic acid (PFNA), perluorodecanoic acid (PFDA), perfluorododecanoic acid (PFDoDA), perfluoroundecanoic acid (PFUnDA), salts of perfluorobutanesulfonate (PFBS), PFHxS, and PFOS were determined by HPLC-ESI-MS/MS. Sample pretreatment, instrumental analysis, quality assurance, and quality control of the analytical method are based on our previous work^22^,^23^,^24^. The detailed pre-treatment of different types of samples, method detection limit (LOD), quality assurance, and quality control are described in the supplementary information.

### Estimated Daily Intake Through Different Pathways and Methods

The daily dietary intake of PFAAs is estimated by the sum of PFAAs in the ingested diet and water. Diet and drinking water consumption are set at 2500 g and 1200 ml per day, respectively. Thus, the daily dietary intake of PFAAs is calculated as equation (1):
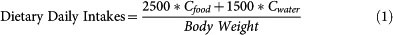
*C_food_* represents concentration of PFAAs in food (ng/g) and *C_water_* stands for concentration of PFAAs in drinking water (ng/mL). According to the questionnaires, the average body weight of local residents was 60 kg (Table S1). The non-dietary exposure of PFAAs was calculated as the sum of dust ingestion and TSP inhalation. The PFAAs daily intake through dust ingestion (DI_dust_) was calculated through the following equation: DI_dust_ = C_dust_ × A_dust_/body weight, where C_dust_ is the concentration of PFAAs in house dust, A_dust_ is the average amount of the daily dust ingestion, and the mean gastrointestinal uptake of dust is set at 50 mg/day for adults according to the US EPA exposure factors handbook^25^. For the calculation of TSP intake, the following equation was applied: DI_TSP_ = C_TSP_*V_air_/body weight, where C_TSP_ is the concentrations of PFAAs in TSP, V_air_ is the daily air volume inhaled by adults and was set at 16.0 m^3^ 25. To be consistent with previous studies of PFAAs exposure assessment, we assumed 100% absorption of PFAAs intake through ingestion and inhalation^15^,^26^. Human exposure to PFAAs through dermal absorption of dust was neglected in this study because only a small fraction (0.048%) of the dose could be absorbed through the skin^27^.

Furthermore, based on a simplified one compartment pharmacokinetic model, we estimated the total estimated daily intake (TEDI) of humans by serum PFAA levels. The model was developed by Andersen et al. and Thompson et al.^28^,^29^, and it have been successfully used in several populations including a Chinese population^30^,^31^,^32^. Briefly, PFAAs levels in the serum are a result of exposure dose (*E*) and the change of PFAAs levels could be described as equation(2):
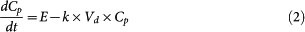
where *k* is the first-order rate constant for PFAAs elimination per day (*k* = 0.693/t_1/2_, and Olsen et al. estimated that the t_1/2_ for PFOA and PFOS were 1257 and 1751 days, respectively, in humans^33^). *V_d_* is the volume of distribution (mL/kg bw). According to previous studies, *V_d_* of PFOA and PFOS were set at 170 and 230 mL/kg bw, respectively^28^,^34^. *C_p_* is the PFAA concentrations in serum. If the serum PFAAs reached steady-state conditions, d*C_p_*/d*t* = 0. Thus, the former equation could be simplified as equation (3):
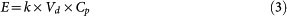


## Results

PFPeA, PFHxA, PFNA, PFDA, PFDoDA and PFUnDA had low detection rates in all samples, and therefore, they were not included for further data analysis and discussion. PFBS, PFHxS, PFOS and PFOA were detected in all serum samples in local residents while PFHpA had a detection frequency of 67%. The average serum PFBS, PFHxS, PFOS, PFHpA, and PFOA concentrations were 22.7, 138, 445, 2.85 and 50.6 ng/mL, respectively. The detailed characteristics of PFAAs in resident demographic subgroups by gender and age (≤45 and >45 years) were provided in Table S2 through S4. The serum PFOS were significantly higher in males than females in local residents(p < 0.05). Compared with males, females have several distinct excretion routes of PFAAs such as menstruation, bearing, and lactation. For example, menstrual clearance rate is even comparable to that renal clearance rate^35^, these particular elimination pathway might result in the lower serum PFOS in females. No significant differences were observed by age in all PFAAs.

PFAAs in drinking waters were relatively low (Table S5), the main PFAAs in drinking waters were PFOS and PFOA and could only be detected in 34% and 56% of samples, respectively. The highest PFOS and PFOA level in drinking water were 3.04 and 2.08 ng/L, respectively, which were lower than that in drinking water reported in previous studies^36^,^37^,^38^. The result indicated that drinking water in this area was not contaminated by PFAAs. PFBS was the most prevalent PFAAs in duplicate diet samples, with an average of 7.02 ng/g ww, followed by PFOS and PFHxS (Table S6). The highest PFOS, PFHxS, and PFOA levels in diet samples were 3.47, 0.45, and 0.10 ng/g ww, with averages of 0.82, 0.19, and 0.06 ng/g ww, respectively. Compared with a study on duplicate diet monitoring in Germany, both the detected rates and levels were higher in this area^39^. The levels of PFAAs in TSP samples collected from the plant (TSP-P) were extremely high, and PFAAs levels decreased sharply from the point source. However, PFAAs levels in TSP samples collected around the plant (TSP-A) were much higher than that in Europe and Japan^40^,^41^, which suggested that the PFAAs might be spread to the surroundings by the TSP route (Table S7). PFOS, PFOA and PFHxS were the major compounds in dust samples, extremely high levels of PFAAs were detected in dust samples collected from the plant (Dust-P). The highest concentrations of PFOS, PFOA and PFHxS in Dust-P reached 3157, 253, and 257 μg/g of dry weight. In dust samples collected around the plant (Dust-A), PFOS, PFOA and PFHxS ranged from 2.68 to 18486 ng/g, 5.00 to 1230 ng/g, and 0.44 to 708 ng/g, with the average of 1189, 354 and 68.0 ng/g, respectively, which were almost three orders of magnitude lower than that in Dust-P.

The daily intake of PFAAs through dietary, dust ingestion, and TSP inhalation were calculated according to equation 1–3(Table 2). Daily dietary intakes of PFBS, PFHxS, PFOS, PFHpA, and PFOA for local residents were 270, 7.32, 31.2, 5.63 and 2.0 ng/day/kg bw, respectively. The DI_dust_ for occupational workers reached 1679 ng/day/kg bw, and the DI_dust_ of PFOS and PFOA were 604 and 759 ng/day/kg body, respectively. These values were almost three orders of magnitude higher than those estimated in previous studies^18^,^26^. The DI_dust_ of PFOS, PFOA, and PFHxS of local residents was 0.991, 0.295, and 0.057 ng/day/kg body, respectively. PFAAs exposure via TSP inhalation was lower than dietary intake and dust ingestion in local residents (Table 2). According to Equation (3), the total estimated daily intake (TEDI) of PFOS and PFOA for local residents was 19.0 and 1.43 ng/day/kg body, respectively.

## Discussion

As a point source, this area provided an ideal platform for us to study the exposure pathways to humans and bioaccumulative behaviour of different PFAAs. The dominance of PFHxS, PFOS, and PFOA in residents around the fluorochemical facility was consistent with the results on environmental samples from the same area^22^. Serum PFAAs of local residents were nearly one order of magnitude higher than that of general populations in China^23^,^30^. Elevated serum PFAAs levels were also found in the nearby community of a fluorochemical industrial zone in northern China. The predominant PFAAs in residents in that study was PFOA (378 ng/mL), which was much higher than that in our study^42^. However, the serum PFOS and PFHxS in their study were comparable to that in the general population and were much lower than our study (PFOS:39.4 vs. 445 ng/mL; PFHxS: 1.12 vs. 138 ng/mL)^42^. This is most likely due to the different production pattern between two plants. The manufacturing plants in their study mainly produced fluoropolymers such as polytetrafluoroethylene, which was known to contain PFOA as a contaminant. The manufacturing plant in the present study primarily focused on PFSA(perfluorinated sulfonic acid)-based chemicals and never produced PFOA.

**Figure 1:**
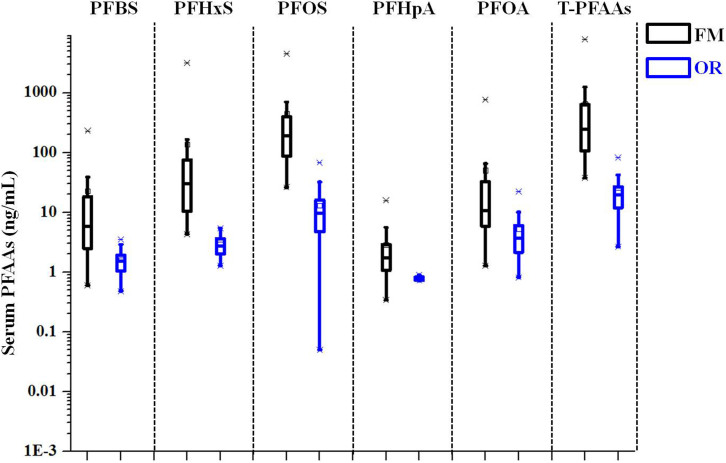
Boxplots of PFAAs concentrations in serum samples of family members of occupational workers (FM group) and ordinary residents (OR group).

Our previous study found a decreasing gradient of PFAAs in soils and hen eggs with increasing distance from the plant^22^, but no such spatial trend was found in the serum of the studied population. The geometric mean serum levels of PFAAs was approximately 17 times higher in the highest quartile (Q1) than that in the lowest quartile (Q4) (1352 vs. 81 ng/mL) (Table S8). We found that serum PFAAs levels in FM group (n = 32) were statistically significantly higher than that in OR group (n = 26) (p < 0.01). The average serum PFAAs in the FM group were 1023 ng/mL, while the average serum PFAAs in the OR group were only 181 ng/mL(Table S9, Figure 1).The significantly different PFAAs body burden between FM and OR suggested that there was a special exposure pathway for PFAAs entering into FM.

The exposure pathways of PFAAs for local residents were further examined in order to find the specific route for FM. There were no obvious difference in the PFAAs level in TSP samples between FM and OR (0.56 ng/m^3^ in the FM group's houses vs. 0.89 ng/m^3^ in the OR group's houses). In contrast to TSP, dust PFAA levels were significantly higher in the FM group (6775 ng/g) than in the OR group (429 ng/g) (p < 0.01). It was surprising that the high PFAA levels in dust seems not play a role in elevating the PFAA levels in TSP via resuspension in this study. It might because that the large noise of the TSP sampler kept human activities (such as housework, walking around the samplers and so on) away during TSP sampling process. High PFAA levels in dust samples collected from FM indicated that occupational workers elevated the PFAAs levels in the living environment. PFAAs levels in the duplicate diet were considerably higher in the FM group than in the OR group, PFOS level in duplicate diet from FM group was 1.41 ng/g ww, while it was only 0.077 ng/g ww in the duplicate diet from OR group. However, the food material of both groups was mainly purchased from the local market, and the drinking water was supplied by municipal water systems, so there was no special food source for the FM group according to questionnaires. Through our observation and further investigation, most of the occupational workers did not change their clothes after work. We collected clothes of both workers and residents, used distilled water to wash them, and then analysed PFAAs in the wash-off water (detailed information in SI). To be convenient for comparison, PFAAs concentrations in wash-off water were normalized to clothes weight according to equation (4):
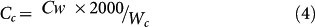
where *C_c_* represents the concentration of extractable PFAAs in the clothes, *C_w_* represents the PFAAs concentrations in wash-off water, and *W_c_* represents the weight of the clothes. The PFAA levels in workers' clothes were significantly higher than that in residents' clothes (p < 0.05), with the GM of 218275 and 4402 ng/g, respectively (Table S10). Although not 100% of PFAAs in clothes were extracted during our washing process, however, the analysis results indicated that PFAAs concentrations in workers' clothes were almost 50 times higher than that in ordinary residents' clothes. Therefore, through the uniform manner of washing process, the determined PFAAs levels in wash-off water reflected the actual PFAAs levels in clothes. This result proved that workers unintentionally carried PFAAs to their residence via their work clothes, leading to elevated PFAAs exposures in their living environments and consequently resulting in higher PFAAs body burden of their family members. This further explained the higher PFAAs levels in the duplicate diet from FM than that in the OR group (in the case that they used the same source of food material). PFAAs probably transferred from contaminated clothes to the dinner via cooking processes, which suggested a potential PFAAs exposure pathway other than direct dusts ingestion. Contaminated clothes – diet - human is a new exposure pathway identified in our study. Such unusual route might occur in similar cases of occupational exposure, and more attention should be paid on it. Moreover, the human exposure via the new pathway could be effectively avoided by compulsory measure of changing work clothes after work.

The total PFAAs daily intake (sum of dietary intake and DI_dust_) of FM was higher than that of OR, which was consistent with the PFAAs body burden. Dietary intake was the major exposure pathway in the present study, which was consistent with previous studies^15^,^18^,^19^,^21^. DI_dust_ of PFOS and PFOA accounted for 5.2% and 20.0% of the total daily intake (TDI) in the FM group, and 6.0% and 8.0% in OR group, respectively. Compared to that DI_dust_ accounted for less than 2% of daily intake in the Chinese population^18^, our result further suggested that dust ingestion was an important exposure pathway to residents around the fluorochemical plant. Moreover, the house dust exposure pathway could contribute to a much higher exposure to toddlers due to the more frequent hand-to-mouth behaviour. Recently studies suggested that some PFAAs were associated with adverse health effects in children^12^,^26^, the health effects of PFAAs exposure to local children need to be further investigated.

In this study, a steady state condition of PFAAs accumulation/elimination in residents was assumed. The TEDI of PFOS and PFOA was slightly lower than TDI; serum PFAAs could be underestimated if the exposure time was not enough to achieve the steady state, possibly resulting in a low TEDI value. Furthermore, the TEDI estimated through this model referred to a daily absorption dose of humans, and in reality, humans would not absorb all PFAAs ingested into the body. In this study, the ratio of TEDI and TDI for PFOS and PFOA were 60.9% and 72.5%, respectively, which might indicate the absorption rate of PFAAs.

**Figure 2:**
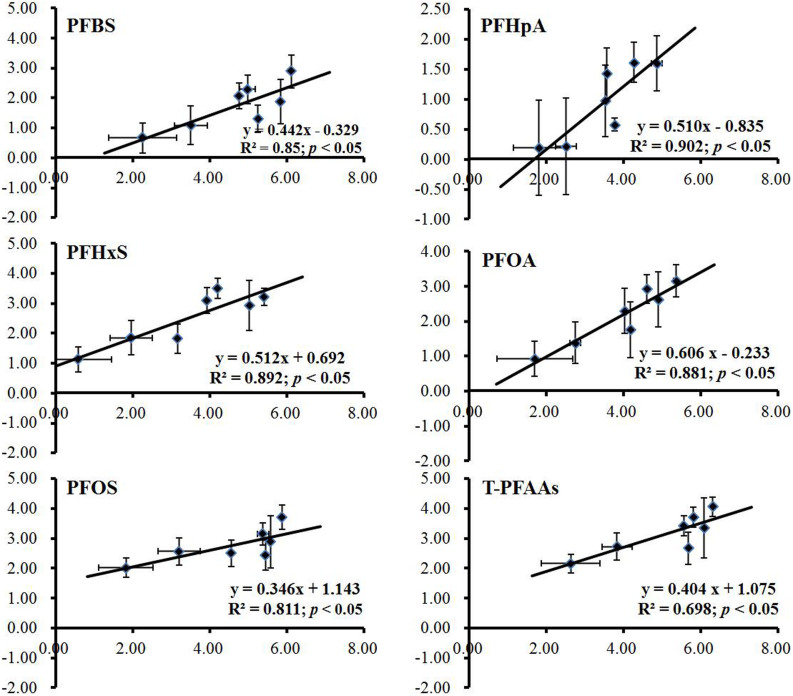
Linear correlations between logarithm PFAAs of serum and dust samples. The X and Y axis represent logarithm serum and dust concentrations of PFAAs, respectively.

The above discussion has illustrated that dust played an important role in this case. The correlation between dust PFAAs levels and serum PFAAs levels were further investigated. There were seven groups of dust data (dust samples from FM, dust samples from OR, and dust samples from the plant were divided into five sub-groups: the sulphonation department, the fabric finishing agent department, the electrolytic department, the research building, and the management office) and two groups of serum data (FM and OR group) in this study. Thus, we cited a set of unpublished data (data only for the reviewer), the serum PFAAs levels of occupational workers from different departments in this plant in 2011 (including workers from management office, electrolytic department, fabric finishing agent department, research building, and sulphonation department), to get more paired data of dust and serum samples and make a better significance of fit. The result indicated that logarithm serum PFAA concentrations were significantly correlated with the corresponding logarithm dust PFAA concentrations (p < 0.05), which suggested that PFAAs body burden was closely related to dust ingestion in this setting (Figure 2).

**Figure 3:**
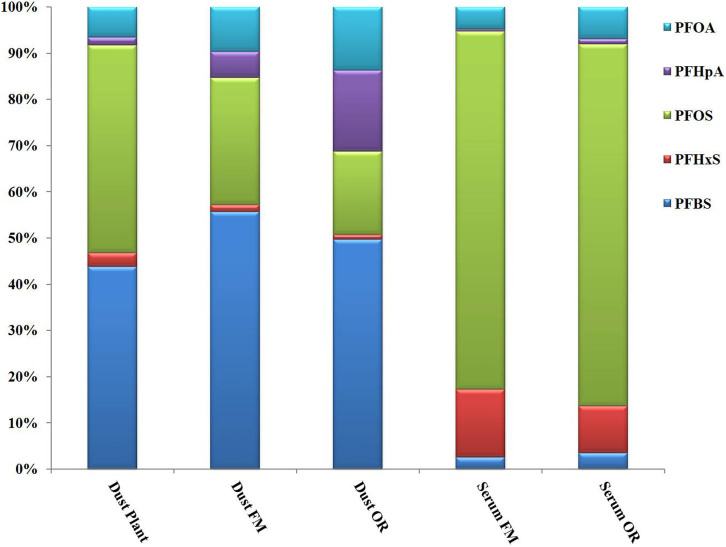
PFAAs composition profiles in different groups of sample matrices.

The PFAAs profiles changed largely between the dust and serum samples of local residents. PFSAs accounted for 68.2% of the total PFAAs in dust samples, which then increased to 87.4% in serum samples in the present study (Figure 3). The results suggested that PFSAs are more preferentially accumulated in humans than perfluorocarboxylic acids (PFCAs), which is also consistent with previous research in animals. As for individual PFAAs, PFBS, PFOS and PFOA accounted for 50.5%, 20.1%, and 15.9% of the total PFAAs in dust samples, respectively. However, PFOS became the predominant PFAAs in serum samples, which accounted for 78.3%, followed by PFHxS (12.6%), whereas the proportion of PFBS sharply decreased from 50.5% in dust to 2.9% in serum.

The ratio of the proportion of PFAA_Serum_ to the proportion of PFAA_Dust_ was calculated to illustrate the different accumulation potential of the studied PFAAs, and this value was defined as the potential bioaccumulative index (PBI) in later discussions. The PBI of five PFAAs were ranked as follows: PFHxS (7.41) > PFOS (3.90) > PFOA (0.359) > PFBS (0.057) > PFHpA (0.051). The PBI_PFHpA_ and PBI_PFBS_ exhibited a low bioaccumulation potential in the present study, and it was consistent with Conder et al.'s report that PFCAs with less than seven fluorinated carbons might not accumulate in biota^8^. The PBI_PFHxS_ and PBI_PFOS_ were nearly 130 and 68.4 times higher than PBI_PFBS_, respectively. Olsen et al. reported that half-lives of PFHxS and PFOS were 107 and 70 times of PFBS, respectively, which were highly consistent with the PBI results in this study^33^,^43^. The PBI of PFHxS suggested the high accumulative capacity in humans, which partly supported the longer half-life of PFHxS than PFOS in human^33^. The bioaccumulation potential of PFOA is still a controversial issue^8^,^33^,^44^. Kowalczyk et al. reported that the kinetics of PFOA were similar to those of PFBS and substantially differed from those of PFHxS and PFOS in dairy cows^45^. We can conclude that both the length of fluorinated carbon chain and the function group influence the bioaccumulation potentital of PFAAs. Long chain PFAAs, PFOS and PFHxS, are more bioaccumulative than PFBS in our case. Although PFHxS and PFOA has six and seven perfluorinated carbons, respectively, the PBI value of PFHxS is much higher than that of PFOA in our study, suggesting that PFSAs are much more bioaccumulative than PFCAs in humans.

## Supplementary Material

Supplementary Information Supplementary information

## Figures and Tables

**Table 1:** Overview of sampling information

Sampling site		Serum	Dust	TSP^a^	DW^b^	DD^c^	Clothes
Plant			8	7			5
			(Dust-P)^d^	(TSP-P)^e^			
Around Plant		58	30	8	6		
			(Dust-A)^f^	(TSP-A)^g^			
	FM^h^	32	9	4	3	5	
	OR^i^	26	21	4	3	4	10

^a^TSP: Total Suspended Particle.

^b^DW: Drinking Water.

^c^DD: Duplicate Diet.

^d^Dust-P: dust samples collected in the plant.

^e^TSP-P: TSP samples collected in the plant.

^f^Dust-A: dust samples collected around the plant.

^g^TSP-A: TSP samples collected around the plant.

^h^FM: Family member of workers.

^i^OR: Ordinary residents.

**Table 2:** Estimated Daily Intake (EDI) of PFAAs via Dust, TSP in this study and Total Estimated Daily Intake(TEDI) calculated by a simplified one compartment pharmacokinetic model (ng/day/kg body)

	OW^a^	LR^b^	FM^c^	OR^d^
Dust ingestion (ng/day/kg body)				
PFBS	921	1.64	4.09	0.593
PFHxS	554	0.057	0.145	0.013
PFOS	604	0.991	2.96	0.148
PFHpA	225	0.179	0.315	0.127
PFOA	759	0.295	0.581	0.158
T- PFAAs	1679	3.16	8.09	1.04
TSP inhalation (ng/day/kg body)				
PFBS	67.3	0.065	0.069	0.068
PFHxS	0.763	0.017	0.015	0.020
PFOS	4.39	0.061	0.058	0.069
PFHpA	33.3	0.120	0.053	0.210
PFOA	296	0.084	0.046	0.132
T- PFAAs	402	0.346	0.24	0.499
Daily Dietary Intake (ng/day/kg body)				
PFBS	444	270	444	52.1
PFHxS	10.0	7.32	10	3.94
PFOS	54.3	31.2	54.3	2.33
PFHpA	8.37	5.63	8.37	2.22
PFOA	2.26	2.01	2.26	1.70
T- PFAAs	519	316	519	62.3
Total Estimated Daily Intake (ng/day/kg body)				
PFOS	1000	19	32.9	9.34
PFOA	374	1.43	2.23	0.792

^a^:OW:occupational workers of the fluorochemical plant;

^b^:LR:local residents (include FM and OR);

^c^: FM: family members of occupational workers;

^d^: OR: ordinary residents (local residents who are not the family members of occupational workers).
