# Supplementary material for: Elevated levels of perfluoroalkyl acids in family members of occupationally exposed workers: the importance of dust transfer
Source: Sci Rep. 2015 Mar 20;5:9313. doi: 10.1038/srep09313 (PMC5380130; doi:10.1038/srep09313)
Supplement: Supplementary Information [file srep09313-s1.pdf]

Supplementary information

Elevated levels of perfluoroalkyl acids in family members of occupationally exposed workers: the importance of dust transfer

Jianjie Fu[1], Yan Gao[1], Thanh Wang[1], Yong Liang[2], Yawei Wang*[1], Aiqian Zhang*[1], and Guibin Jiang[1]

[1]State Key Laboratory of Environmental Chemistry and Ecotoxicology, Research Center for Eco-Environmental Sciences, Chinese Academy of Sciences, Beijing 100085, China

[2]Jianghan University, Wuhan, Hubei, China

*Corresponding author

Prof. Aiqian Zhang

Tel: 8610-62849157

Fax: 8610-62923549

E-mail: aqzhang@rcees.ac.cn

Dr. Yawei Wang

Tel: +8610-62849334

Fax: +8610-62849339

E-mail: ywwang@rcees.ac.cn

Research Center for Eco-Environmental Sciences,

Chinese Academy of Sciences

Sampling Procedure

A total of 38 dust samples were collected; 8 dust samples were from different departments of the fluorochemical plant (Dust-P) and 30 dust samples from around the plant (Dust-A). Fifteen TSP samples were also collected, including 7 TSP samples from different departments of the plant (TSP-P) and 8 TSP samples in the surrounding environment around the fluorochemical plant (TSP-A). The spatial distribution of the sampling sites is shown in Figure S1.

Fifty-eight local residents who lived around this plant within a distance of less than 3 km were recruited in present study. Approximately 10 mL of blood sample was collected from each participant by a vacuum blood collection tube. The serum was separated from clotted blood by centrifugation at 1100 × g at 4 °C for 10 min and transferred to a 5mL corning polypropylene tube. The serum samples were placed on ice packs and immediately sent to the laboratory. The dust samples were collected by sweeping plastic brushes on the surface of the furniture. The TSP samples were collected by a mid-volume air sampler (Tianhong Intelligent Instrument Plant, Wuhan, China); the flow rate was set at 120 L per minute for 24 hours per sample. The TSP was collected on a Whatman quartz fibre filter (QFF), which was weighed before and after sampling to determine the TSP concentration. Both the dust and TSP samples were wrapped in aluminium foil and sealed in small polyethylene zip bags. All of the samples were stored at −20 °C until analysis.

According to our questionnaires, the food source of our studied populations was mainly directly purchased from the local market, and the drinking water was supplied by the municipal water systems. During our sampling time, we collected 6 drinking water samples directly from the tap and 9 duplicate diet samples from local residents. The diet samples were directly collected from their table when they were eating their meals. The diet samples were stored in a cooler with ice packs during the sampling day and transported to the laboratory where they were then homogenised and lyophilised. Furthermore, drinking water samples were directly collected from tap

waters from municipal water systems. Clothes were randomly collected from five workers when they went home from work and nine clothes of nearby residents were also collected.

Sample Pretreatment

Serum, dust, TSP samples

The serum samples were first spiked with 5 ng mass-labelled PFOS and PFOA and then extracted by ion-pairing extraction using MTBE three times. The combined extraction was further concentrated under gentle nitrogen gas and the solvent exchanged with 0.5 mL methanol. The mixture was then diluted with 50 mL water and loaded onto an Oasis-HLB cartridge (Waters, 6 cc, 150 mg). The cartridge was washed using 5 mL of methanol/water (1:4) after the sample was loaded. The target analytes were then eluted using 10 mL of methanol. The final elution was concentrated to 1 mL for HPLC-MSMS analysis. For TSP samples, the filters were cut into small pieces (less than 2×2 mm) by a pair of stainless steel-scissors before extraction. The dust and TSP samples were first spiked with 5 ng mass-labelled internal standards. After equilibrating for approximately four hours, 5 mL of methanol was added to extract PFAAs, samples were extracted under ultrasound for 20 minutes, the ultrasonic extraction was performed for three times. The extracts were combined and concentrated to 0.5 ml under gentle nitrogen gas. The solid phase extraction procedure was then performed in the same way as the serum samples.

Drinking water samples and the wash-off water of clothes

Solid phase extraction was used for water samples. The WAX column (Waters, 6 cc, 150 mg) was pre-activated using 4 mL of 0.1 % ammonium hydroxide in methanol, 4 mL of methanol and 4 mL of water. Before loading, 5 ng of internal standards ^{13}C-labelled PFHxS, PFOS, and PFOA were added into the 500 ml water sample and equilibrated for two hours. After 500 ml water sample was loaded on the column, four

millilitres of ammonium acetate (pH=4) was loaded. Then, the column was dried by centrifugation at 3000 rpm for 15 min. The WAX column were cleaned by 4 mL of methanol to remove the impurity after drying, the target compounds were eluted by 4 mL of 0.1 % ammonium hydroxide in methanol. Finally, the elution was concentrated to approximately 1 ml using a gentle stream of N_2.

The clothes were first weighed and placed into a polypropylene box. Two litres of distilled water was added to the box and shaken for approximately 20 min. The wash-off water was collected to analyse the PFAAs. We took 100 ml of the wash-off water to analyse the PFAAs and the pretreatment procedure for the wash-off water was the same as that for the drinking water except for the sampling volume.

Duplicate diet samples

Approximately 0.2g of the diet dried samples was weighed into the PP bottles. Ten millilitres of 50 mM KOH in methanol was added and shaken for 16 hours. The extract was concentrated to 1 mL. The concentrated extract was added with 40 mL of Milli-Q water and 0.5 mL of 1 M HCl. The WAX cartridges were preconditioned with 4 mL of 0.1 % ammonium hydroxide in methanol, 4 mL of methanol and 4 mL of water. The diluted extract was loaded onto the cartridge. Four millilitres of ammonium acetate (pH=4) was then added. Then, the column was dried by centrifugation at 3000 rpm for 15 min. The cartridges were cleaned with 4 mL of methanol and then eluted with 4 mL of 0.1% ammonium hydroxide in methanol. The elution was concentrated to 1 mL for injection.

Supplementary Results

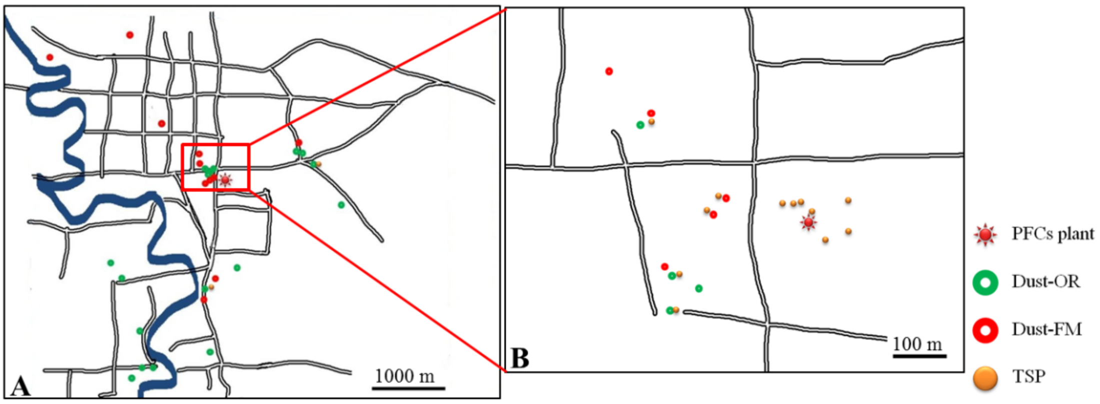

Figure S1 The spatial distribution of dust and TSP sampling sites.

(Dust-OR: dust samples from ordinary residents; Dust-FM: dust samples from a family member of occupational workers)

Table S1.Descriptive statistics of the demographic information in this study

		Min	Max	Median	Average	SD
	Height (cm)	150	180	164	163	7
Total	Weight (kg)	42	80	57	60	10
58	BMI (kg/m^2)	17.2	28.0	22.3	22.2	2.8
	Age	21	71	48	48	13
Female	Height (cm)	150	170	160	160.5	5.0
41	Weight (kg)	42	75	55	55.9	8.6
	BMI (kg/m^2)	17.2	28.0	21.8	21.7	2.8
	Age	21	71	47	47.5	12.9
Male	Height (cm)	154	180	170	170	6
17	Weight (kg)	52	80	65	68	9
	BMI (kg/m^2)	18.9	26.7	23.9	23.5	2.4
	Age	22	65	49	50	12

Table S2. Spiked recoveries of PFAAs in different matrices (n=3)

Spiked-concentration		PFBS	PFHxS	PFOS	PFHpA	PFOA
(ng/g)				GFF filter		
	Recovery	99.2%	95.5%	93.0%	99.9%	112.6%
5	RSD	2.3%	2.4%	2.0%	3.0%	2.8%
	Recovery	105.5%	107.0%	110.5%	93.8%	105.5%
10	RSD	3.5%	5.7%	6.4%	11.5%	6.4%
(ng/g)				Dust		
	Recovery	85.1%	75.9%	88.3%	76.1%	81.9%
10	RSD	7.3%	2.1%	6.9%	7.8%	9.0%
	Recovery	90.9%	87.5%	86.8%	87.0%	80.6%
50	RSD	1.8%	1.7%	1.7%	1.7%	1.6%
(ng/mL)				Serum		
25	Recovery	95.5%	110.3%	101.5%	82.6%	96.6%
	RSD	3.5%	4.2%	3.8%	1.6%	3.1%
50	Recovery	102.8%	105.7%	98.1%	84.2%	94.9%
	RSD	14.6%	1.4%	2.4%	1.7%	4.4%
(ng/g)				Duplicate diet		
0.5	Recovery	91.5%	98.9%	99.0%	102.7%	96.3%
	RSD	10.4%	8.4%	3.2%	17.9%	4.9%
5	Recovery	85.3%	82.2%	90.2%	63.2%	98.1%
	RSD	6.3%	4.6%	5.9%	7.3%	4.0%
(ng/L)				Water		
10	Recovery	78.7%	106.4%	85.6%	81.5%	89.9%
	RSD	5.9%	4.9%	4.3%	3.3%	9.3%
100	Recovery	104.9%	111.3%	110.0%	102.6%	107.7%
	RSD	3.1%	7.9%	10.2%	5.9%	0.6%

Table S3.Descriptive statistics for serum PFAAs in local residents (n=58, ng/mL)

	Min	Max	Median	AM	GM[a]
PFBS	0.6	230	6.4	22.4	7.85
PFHxS	4.33	3164	27.6	133.9	33.7
PFOS	26.1	4503	196.6	438.1	210
PFHpA	n.d.	15.7	1.1	2.0	1.9
PFOA	1.28	765	10.7	49.6	15.2
Total PFAAs	37.4	7830	257.8	645.8	299

a:In cases where an individual PFAA was not detected, the levels were set as LOD/2 during the calculation of the geometric mean.

Table S4.Detailed descriptive statistics for serum PFAAs (ng/ml) in local residents divided by males and females

	Min	Max	Median	AM	GM	Min	Max	Median	AM	GM
	Male (n=21)					Female (n=37)				
PFBS	0.84	129	9.6	26.7	9.5	0.6	230	6.05	20.6	7.04
PFHxS	4.33	3164	75.02	355	51.5	5	236	25.5	42.3	26.5
PFOS	53.5	4503	245.75	920	326	26.1	956	176	238	164
PFHpA	0.77	15.74	1.065	2.13	2.73	n.d.	7.7	1.14	1.92	1.59
PFOA	1.94	765	11.52	108	20.8	1.28	142	10.6	25.4	12.8
Total PFAAs	92.8	7830	625.44	1411	464	37.4	1243	242	328	233

Table S5.Detailed descriptive statistics for serum PFAAs (ng/ml) in local residents divided by age

	Min	Max	Median	AM	GM	Min	Max	Median	AM	GM
			≤ 45 (n=23)					> 45 (n=35)		
PFBS	0.72	121	4.00	17.2	7.22	0.6	230	10.5	26.3	8.29
PFHxS	5	3164	25.5	73.1	39.6	4.33	498	29.1	180	30.3
PFOS	36	4503	199	362	185	26.1	3014	189	496	229
PFHpA	n.d.	7.7	1.30	2.21	1.39	0.76	15.7	1.10	1.80	2.27
PFOA	1.28	765	10.7	50.2	13.7	1.94	505	10.6	49.1	16.4
Total PFAAs	57.2	7830	247	505	276	37.4	3649	268	753	314

Table S6. PFAAs in drinking water (ng/L)

	PFBS	PFHxS	PFOS	PFOA	PFHpA
OR1	1.62	2.56	n.d.	2.08	n.d.
OR2	1.64	n.d.	n.d.	2.02	n.d.
OR3	9.52	n.d.	n.d.	n.d.	n.d.
FM1	6.54	n.d.	n.d.	n.d.	n.d.
FM2	6.56	n.d.	n.d.	n.d.	n.d.
FM3	37.4	3.50	3.04	n.d.	2.98

Table S7. PFAAs in the duplicate diet samples (ng/g ww)

	PFHpA	PFOA	PFBS	PFHxS	PFOS
		Duplicate diet of local residents			
1	n.d.	n.d.	n.d.	0.119	n.d.
2	n.d.	n.d.	n.d.	0.101	n.d.
3	n.d.	n.d.	n.d.	0.123	n.d.
4	0.18	n.d.	5.247	0.067	0.158
5	0.513	n.d.	21.167	0.323	2.84
6	0.499	0.103	26.967	0.448	3.47
7	n.d.	n.d.	3.837	0.118	0.247
8	n.d.	n.d.	1.447	0.276	0.21
9	n.d.	n.d.	4.297	0.138	0.294
GM	0.10	0.05	1.28	0.16	0.24
AM	0.17	0.06	7.01	0.19	0.82
SD	0.20	0.02	9.97	0.13	1.34
		OR			
1	n.d.	n.d.	n.d.	0.119	n.d.
2	n.d.	n.d.	n.d.	0.101	n.d.
3	n.d.	n.d.	n.d.	0.123	n.d.
4	0.18	n.d.	5.247	0.067	0.158
GM	0.069	0.050	0.160	0.100	0.067
AM	0.083	0.050	1.349	0.103	0.077
SD	0.065	0.000	2.599	0.026	0.054
		FM			
5	0.513	n.d.	21.167	0.323	2.84
6	0.499	0.103	26.967	0.448	3.47
7	n.d.	n.d.	3.837	0.118	0.247
8	n.d.	n.d.	1.447	0.276	0.21
9	n.d.	n.d.	4.297	0.138	0.294
GM	0.13	0.06	6.71	0.23	0.68
AM	0.23	0.06	11.54	0.26	1.41
SD	0.25	0.02	11.67	0.14	1.61

Table S8.Detailed information of PFCs levels in dust and TSP samples (Dust: ng/g; TSP: ng/m^3)

	Min	Max	GM	Min	Max	GM	Min	Max	GM
	Dust-P			Dust-A			Dust-FM		
PFBS	57293	5338491	329766	4.69	13854	433	1165	13854	3234
PFHxS	1481	257201	22666	0.435	708	14.5	15.4	708	91.4
PFOS	36212	3156673	337715	2.68	18487	173	271	18487	1599
PFHpA	3415	92674	12583	1.84	825	101	104	825	323
PFOA	10901	253081	49870	5.00	1230	136	63.4	1230	566
	Dust-OR			TSP-P			TSP-A		
PFBS	4.69	3174	183.1	0.533	1708	8.15	0.024	0.486	0.154
PFHxS	n.d	91.0	3.84	0.439	13.7	1.44	0.014	0.215	0.040
PFOS	2.68	688	66.6	0.498	93.6	3.54	0.021	0.608	0.132
PFHpA	1.84	632	64.9	1.74	564	17.1	0.006	2.30	0.111
PFOA	n.d	793	50.7	2.81	5761	75.6	0.022	1.09	0.161
	TSP-FM			TSP-OR					
PFBS	0.024	0.486	0.167	0.030	0.470	0.142			
PFHxS	0.014	0.120	0.039	0.014	0.215	0.041			
PFOS	0.021	0.550	0.128	0.026	0.608	0.136			
PFHpA	0.008	0.612	0.060	0.006	2.30	0.205			
PFOA	0.022	0.379	0.105	0.027	1.091	0.248			

Table S9. Detailed statistics data of serum PFAAs levels in four individual quartiles (ng/mL)

	Q1			Q2			Q3			Q4		
	Min	Max	GM	Min	Max	GM	Min	Max	GM	Min	Max	GM
PFBS	3.65	230	27.2	1.38	114	8.62	0.840	32.8	6.55	0.600	18.4	2.46
PFHxS	29.1	3164	178	11.0	236	42.3	6.00	52.6	22.4	4.33	31.7	7.57
PFOS	201	4503	889	174	432	266	72.3	220	137	26.1	92.3	59
PFHpA	1.36	11.1	3.53	0.793	15.7	2.23	0.853	2.66	1.34	n.d.	1.74	0.860
PFOA	8.36	765	54.9	5.84	80.5	16.5	1.94	102	10.8	1.28	34.5	5.42
Total PFAAs	625	7830	1352	268	579	367	110	248	195	37.4	108	81.0

Table S10.Detailed descriptive statistics for serum PFAAs in family members and ordinary residents (ng/ml)

Compounds	Family Members (N=32)					Ordinary Residents (N=26)				
	min	max	median	AM	GM	min	max	median	AM	GM
PFBS	1.38	230	12.9	34.3	12.7	0.60	32.8	4.18	7.64	4.35
PFHxS	11.0	3164	58.9	225	74.3	4.33	155	9.45	21.8	12.7
PFOS	103	4503	319	686	390	26.1	591	82.8	133	98.1
PFHpA	0.79	11.1	1.84	3.10	2.26	n.d.	15.7	1.50	2.34	1.48
PFOA	4.3	765	19.2	75.8	24.2	1.28	102	7.81	17.2	8.62
T-PFAAs	144	7830	443	1023	564	37.4	774	105	181	136

Table S11.Attached PFAAs levels on clothes of workers and nearby residents (ng/g)

No.	PFOS	PFBS	PFHxS	PFHpA	PFOA	Total
Ordinary residents						
1	2035	10531	431	190	113	13300
2	582	6578	197	52.7	32.1	7441
3	598	3115	38.1	40.2	25.6	3816
4	1557	4148	28.0	22.6	23.4	5779
5	30.0	153	14.2	76.6	18.3	292
6	97.0	6718	50.9	357	157	7381
7	1266	15667	420	2249	2034	21637
8	46.5	1531	18.2	127	41.4	1763
9	42.2	3281	25.4	71.2	43.6	3463
GM	271	3466	61.5	120	65.7	4402
Occupational workers						
10	28185	95810	6771	1374	1611	133750
11	15882	54534	1425	396	657	72893
12	36918	175483	3024	2208	3000	220633
13	271021	662595	14954	4425	5070	958065
14	91581	136986	3849	3133	4872	240422
GM	52795	152773	4416	1755	2392	218275
